# Supplementary material for: A Cutoff Determination of Real-Time Loop-Mediated Isothermal Amplification (LAMP) for End-Point Detection of Campylobacter jejuni in Chicken Meat
Source: Vet Sci. 2022 Mar 8;9(3):122. doi: 10.3390/vetsci9030122 (PMC8953776; doi:10.3390/vetsci9030122)
Supplement: Supplementary file 1 [file vetsci-09-00122-s001.zip › vetsci-1587973-supplementary.pdf]

veterinary sciences
MDPI

Supplementary Materials

```
proc logistic data=roc1;
    model positive(event='1')=cycle/outroc=rocdata;
    roc; roccontrast;
run;

data roc2; set rocdata;
    logit=log(_prob_/(1-_prob_));
    Cutoff=(logit-2.3689)/-0.0682;
    Sensitivity=_sensit_;
    Specificity=1-_1mspec_;
    YJ=Sensitivity+Specificity-1;
run;

proc sort data=roc2;
    by descending YJ;
run;

proc print data=roc2 (obs=25) noobs;
    var Cutoff Sensitivity Specificity YJ;
run;
```

Figure S1. SAS code for the ROC curve analysis

Table S1. A 2 × 2 table for calculation of sensitivity, specificity, and predictive values of the LAMP assay

	Culture positive	Culture negative	Total
LAMP positive	31	2	33
LAMP negative	1	46	47
Total	32	48	80
